# Differences in cognitive side-effects and seizure parameters between thiopental and propofol narcosis in ECT

**DOI:** 10.1192/j.eurpsy.2021.2071

**Published:** 2021-08-13

**Authors:** E. Kavakbasi, A. Wieneke, B. Baune

**Affiliations:** 1 Department Of Psychiatry And Psychotherapy, University of Münster, Münster, Germany; 2 Department Of Psychiatry, Melbourne Medical School, Melbourne, Australia; 3 The University Of Melbourne, The Florey Institute of Neuroscience and Mental Health, Parkville, Australia

**Keywords:** ect narcosis, thiopental, Electroconvulsive therapy, propofol

## Abstract

**Introduction:**

The standard anaesthetic for electroconvulsive therapy (ECT) in our hospital methohexital was no longer available from the beginning of 2019. A change to alternatives became necessary. We initially decided on thiopental and than switched to propofol after the suspicion of increased occurrence of cognitive deficits due to thiopental narcosis was expressed by clinicians.

**Objectives:**

This retrospective study provides a comparison of the two narcotics in terms of side-effects and seizure parameters.

**Methods:**

We performed a retrospective data collection from our clinical database and identified a total of 64 patients (w=60.9 %, m=39.1 %) got either thiopental (n=35) or propofol (n=29) for ECT narcosis.

**Results:**

The mean age at the beginning of the ECT series was 56.0 years (20-82, SD 17.8, median 57.5). The groups did not differ in terms of age distribution. On average the depressive episode lastet for 9.0 months (SD 11.5, median 6.0) with no difference between the two groups. The mean EEG seizure time was significantly shorter in the propofol group (28.1 sec; 95%-CI: 23.8-32.4) than in the thiopental group (38.3 sec.; 95%-CI: 34.3-38.3). The mean EMG seizure activity was also shortler in the propofol group (12.0 sec.; 95%-CI: 8.0-15.0) compared with the thiopental group (21.5 sec.; 95%-CI: 18.3-24.8). The ECT series was interrupted due to cognitive side-effects in 20 cases. The majority of these cases (n=17) concerned the thiopental group, compared to 3 cases in the propofol group.

**Conclusions:**

Propofol narcosis in ECT was assiosiated with worse seizure parameters, whereas thiopental narcosis was associated with increased risk of cognitive side-effects.

**Disclosure:**

No significant relationships.

